# Early Draper-mediated glial refinement of neuropil architecture and synapse number in the Drosophila antennal lobe

**DOI:** 10.3389/fncel.2023.1166199

**Published:** 2023-06-02

**Authors:** Darren A. Jindal, Hans C. Leier, Gabriela Salazar, Alexander J. Foden, Elizabeth A. Seitz, Abigail J. Wilkov, Jaeda C. Coutinho-Budd, Heather T. Broihier

**Affiliations:** ^1^Department of Neurosciences, Case Western Reserve University School of Medicine, Cleveland, OH, United States; ^2^Department of Neuroscience, University of Virginia School of Medicine, Charlottesville, VA, United States

**Keywords:** glia, synapse, pruning, remodeling, critical period, Draper, antennal lobe, Drosophila

## Abstract

Glial phagocytic activity refines connectivity, though molecular mechanisms regulating this exquisitely sensitive process are incompletely defined. We developed the Drosophila antennal lobe as a model for identifying molecular mechanisms underlying glial refinement of neural circuits in the absence of injury. Antennal lobe organization is stereotyped and characterized by individual glomeruli comprised of unique olfactory receptor neuronal (ORN) populations. The antennal lobe interacts extensively with two glial subtypes: ensheathing glia wrap individual glomeruli, while astrocytes ramify considerably within them. Phagocytic roles for glia in the uninjured antennal lobe are largely unknown. Thus, we tested whether Draper regulates ORN terminal arbor size, shape, or presynaptic content in two representative glomeruli: VC1 and VM7. We find that glial Draper limits the size of individual glomeruli and restrains their presynaptic content. Moreover, glial refinement is apparent in young adults, a period of rapid terminal arbor and synapse growth, indicating that synapse addition and elimination occur simultaneously. Draper has been shown to be expressed in ensheathing glia; unexpectedly, we find it expressed at high levels in late pupal antennal lobe astrocytes. Surprisingly, Draper plays differential roles in ensheathing glia and astrocytes in VC1 and VM7. In VC1, ensheathing glial Draper plays a more significant role in shaping glomerular size and presynaptic content; while in VM7, astrocytic Draper plays the larger role. Together, these data indicate that astrocytes and ensheathing glia employ Draper to refine circuitry in the antennal lobe before the terminal arbors reach their mature form and argue for local heterogeneity of neuron-glia interactions.

## 1. Introduction

Synapse addition and removal must be in proper equilibrium to correctly achieve adult neuronal circuitry. However, when and how synapse number is refined in the central nervous system (CNS) is unclear. Beyond supportive roles in neuronal growth and metabolism (Eroglu and Barres, 2010; Nave, 2010; Kettenmann et al., 2013), glia monitor and modify neuronal circuits (Chung and Barres, 2012; Schafer and Stevens, 2013; Wu et al., 2015; Lee and Chung, 2019). For instance, glial-dependent synapse pruning has been characterized in the neuromuscular junction, cerebellum, dorsolateral geniculate nucleus, hippocampus, and primary visual cortex and is largely mediated by microglia and astrocytes (Neniskyte and Gross, 2017). Four Drosophila glial subtypes: cortex glia, ensheathing glia, astrocytes, and subperineurial glia together share the roles of mammalian microglia and astrocytes (Freeman and Doherty, 2006; Freeman, 2015; Kremer et al., 2017; Pogodalla et al., 2022; Corty and Coutinho-Budd, 2023). Here, we seek to further develop the Drosophila model system to examine the genetic basis of CNS synapse refinement by glia. With well-defined, stereotyped neuroanatomy, independent genetic access to neuronal and glial subtypes, and an extensive library of RNAi constructs and genetic nulls, Drosophila is an ideal model to define pathways underlying synapse-glia interactions. Understanding glia-synapse interactions is important given that dysfunctional signaling contributes to a host of neurological conditions including ASD, epilepsy, schizophrenia, Alzheimer’s disease, Parkinson’s disease, FTD, and noise-induced cochlear damage (Lui et al., 2016; Kim et al., 2017; Neniskyte and Gross, 2017; Wood and Zuo, 2017; Frye et al., 2019; Lee and Chung, 2019; Zhou et al., 2019).

Across vertebrate and invertebrate systems, glia engulf unwanted synapses, though the molecular mechanisms underpinning this key step of neural circuit refinement are largely obscure (Marin et al., 2005; Doherty et al., 2009; Chung et al., 2013; Tasdemir-Yilmaz and Freeman, 2014; McLaughlin et al., 2019). In Drosophila, the primary glial phagocytosis receptor is Draper which has considerable homology to mammalian Jedi-1 and MEGF10, as well as CED-1 in *C. elegans* (Zhou et al., 2001; Suzuki and Nakayama, 2007; Wu et al., 2009). Drosophila glia require Draper to phagocytose neuronal debris (Freeman et al., 2003; Awasaki et al., 2006; MacDonald et al., 2006). Cortex glia engulf dying neurons during development, astrocytes prune mushroom body γ neurites during metamorphosis, and ensheathing glia phagocytose degenerating axonal debris after injury, all in a Draper-dependent manner (Marin et al., 2005; Doherty et al., 2009; Tasdemir-Yilmaz and Freeman, 2014; McLaughlin et al., 2019; Herrmann et al., 2022). However, it is not yet clear whether Draper sculpts neurites and synapses in the CNS outside of the contexts of early metamorphosis and injury. To begin to address this key question, we focused on Draper function in the two glial subtypes that are in close contact with the synaptic neuropil, ensheathing glia and astrocytes. Ensheathing glia normally surround the synaptic neuropil and wrap individual glomeruli, while astrocytes ramify extensively within individual glomeruli (Freeman and Doherty, 2006; Muthukumar et al., 2014; Freeman, 2015; Kremer et al., 2017; Wu et al., 2017; Pogodalla et al., 2022).

We chose the olfactory circuit, specifically the antennal lobe, to probe neuron-glia interactions for its extensive glial infiltration, established behavioral and functional assays, and many shared anatomical and functional similarities to the mammalian olfactory bulb (Vosshall and Stocker, 2007). A given olfactory receptor neuron (ORN), with its cell body in either the antennae or maxillary palps, is activated by an odor that binds and activates its uniquely expressed olfactory receptor (Vosshall and Stocker, 2007). In the antennal lobe, approximately 1,300 cholinergic input ORNs synapse with output projection neurons as well as interneurons in about fifty distinct glomeruli each with stereotyped synapse numbers (Guven-Ozkan and Davis, 2014; Gaudry and Schenk, 2018). Glomeruli can be assigned functions based on the classes of odors which activate the ORN subtype that provides presynaptic input to each. In this work, we chose two glomeruli, VC1 and VM7, for their different odor response profiles. ORNs of subtype pb1A provide input to the VM7 glomerulus and uniquely express the odorant receptor (Or) gene Or42a, while those that synapse onto VC1, of subtype pb2A, coexpress Or85e and Or33c, with a stronger response from Or85e (Goldman et al., 2005). In VC1, the pb2A ORNs can be most intensely activated by the odorant (1R)-(-)-fenchone, a monoterpenoid produced by plants and yeast with antibacterial properties (Dugelay et al., 1992; Carson et al., 2002; Carrau et al., 2005; Soković et al., 2010; Kazemi et al., 2012; Münch and Galizia, 2016; Surburg and Panten, 2016). In VM7, the fruit volatile propyl acetate, also produced by yeasts that Drosophila eat, is the compound at the top of the pb1A ORN odor response profile (Antonelli et al., 1999; Tsakiris et al., 2010; Becher et al., 2012; El Hadi et al., 2013; Christiaens et al., 2014; Zhang and Chen, 2014; Scheidler et al., 2015; Münch and Galizia, 2016).

Here we tested if circuits responsible for transmitting “survival” and “food” information have similar requirements for glial refinement via Draper-mediated engulfment by comparing its functions regulating ORN terminal arbor size and presynaptic content in VC1 and VM7. We developed a semi-automated Imaris pipeline to quantify the effect of *drpr* knockdown in ensheathing glia or astrocytes on axon terminal size, morphology, and presynaptic content in VC1 and VM7. To test whether Draper function is age-dependent, we assessed phenotypes in young adults [3 days post eclosion (DPE)] and in early middle age (12 DPE). We report four major findings. First, glial Draper limits both the terminal arbor size and presynaptic content of VC1 and VM7 ORNs in uninjured animals, arguing for a general requirement for Draper in refining antennal lobe circuitry. To our knowledge, this is the first demonstration of a requirement for glial Draper in refining neurites and synapses in the uninjured antennal lobe. Second, we find clear evidence that Draper limits terminal arbor size and presynaptic content during pupal development or in early adulthood, when individual glomerular volumes are still increasing (this work; Aimino et al., 2023). These data argue that synapse addition and refinement occur simultaneously in this circuit, in contrast to the commonly accepted model that during development, a phase of synapse addition is followed by a phase of synapse removal. Third, astrocytes in the antennal lobe express high levels of Draper in late pupal stages and then rapidly downregulate it in the adult, while Draper expression in ensheathing glia is relatively constant over time. Finally, Draper is required in different glial subtypes for refining ORN terminals in VC1 and VM7. Draper is required primarily in ensheathing glia to restrain terminal arbor size and presynaptic content of VC1; while in VM7, it is required in astrocytes. These data demonstrate that Draper-dependent signaling in both ensheathing glia and astrocytes limits the size of presynaptic terminals in the Drosophila CNS.

## 2. Materials and methods

### 2.1. Animals

Ethical review and approval was not required for this study on *Drosophila melanogaster* in accordance with local legislation and institutional requirements. Flies used in this work were fed a standard molasses diet and were raised at 25°C, 60% relative humidity in incubators (Percival Scientific) on a 12:12 h light:dark cycle. The number of parental flies were optimized to ensure that F1 flies used in experiments were raised in similar concentrations across genotypes. The adult age of flies used is specified in each figure and is measured in days post eclosion (DPE). Male flies were used in all experiments. To fluorescently label the membranes of ORNs, we used Or42a-mCD8::GFP (Couto et al., 2005) for pb1A (gift from K. Broadie, Vanderbilt) and Or85e-mCD8::GFP (Couto et al., 2005) for pb2A (gift from M. Freeman, OHSU). To drive expression in specific glial subtypes, we used MZ0709-Gal4 (Ito et al., 1995) for ensheathing glia (gift from M. Freeman, OHSU) and R86E01-Gal4 (Jenett et al., 2012) for astrocytes (gift from M. Tabuchi, CWRU). We used additional drivers to visualize glial processes: GMR56F03-Gal4 (Jenett et al., 2012) for ensheathing glia (BDSC 39157) and GMR25H07-Gal4 (Jenett et al., 2012) for astrocytes (BDSC 49145). In combination with the drivers above, we used UAS-mCD8::mRFP (Ye et al., 2007) (BDSC 27398), UAS-mCD8::GFP (Pfeiffer et al., 2010) (BDSC 32186), and UAS-mCD8::mCherry (Stork et al., 2014) (gift from M. Freeman, OHSU) to visualize glial membranes. For *drpr* knockdown, we used UAS-*drpr* RNAi (Perkins et al., 2015) (BDSC 36732). This *drpr* RNAi line has been used extensively in the field (Shen et al., 2016; McLaughlin et al., 2019; Chakrabarti and Visweswariah, 2020; Ackerman et al., 2021; Li et al., 2021). When comparing glial infiltration between control and *drpr* knockdown animals, UAS-Luciferase (Perkins et al., 2015) was also in genetic background of control animals to account for Gal4 dilution (BDSC 35788). Mi{PT-GFSTF.1}drpr^MI07659–GFSTF.1^ was used to visualize endogenous Draper expression (Nagarkar-Jaiswal et al., 2015) (BDSC 63184). To drive expression of Brp-short, we used Or85e-Gal4 (gift from J. Carlson, Yale). To express Brp-short, we used UAS-Brp-short-mCherry (Schmid et al., 2008).

### 2.2. Immunohistochemistry

All steps were performed at room temperature unless otherwise noted. For all experiments incorporating Bruchpilot (Brp) staining, flies were fixed for dissection within zeitgeber time 0–2 (2 h after lights on). A control genotype was included with every experiment and was treated identically to knockdown animals. Flies were decapitated and heads fixed in 4% PFA (Thermo Scientific 28906) in PBS with 1% Triton X-100 (PTX10), freshly made for each experiment, for 20 min. Heads were then washed 4 times in PTX10 to remove fix. Fly brains were extracted from heads with forceps, then the visible tracheal network was carefully removed, and then brains were fixed again in 4% PFA in PTX10 for 20 min. To ensure that each brain had the same wait time before fix, each brain was fixed immediately after its dissection, individually. Each brain was then washed in a bulk volume of PTX10 to remove fix and placed in fresh PTX10. After accumulating all brains of a genotype, brains were blocked for 30 min in 5% normal goat serum (Sigma-Aldrich G9023) in PBS with 0.3% Triton X-100. Brains were then incubated in a primary antibody cocktail of mouse anti-Brp (DSHB nc82) and rabbit anti-GFP (Abcam 6556), diluted 1:50 and 1:600, in PBS with 0.1% Triton X-100 (PTX), or a cocktail additionally including rat anti-mCherry (Invitrogen M11217), diluted 1:500, or solely with rat anti-mCherry for 2–3 days at 4°C. The primary antibody cocktail was then removed and PTX10 added. Brains were immediately washed 3 more times for 10 min each on a shaker. Brains were incubated in a secondary antibody cocktail of goat anti-rabbit 488 (Invitrogen A11034) and goat anti-mouse 647 (Invitrogen A32728), diluted 1:300, in PTX, or a cocktail additionally including goat anti-rat 568 (Invitrogen A11077), diluted 1:300, or solely with goat anti-rat 568 for 2–3 days at 4°C. Brains were washed identically as after primary antibody incubation, then mounted whole as previously described (Wu and Luo, 2006). For Draper expression experiments, flies expressing GMR56F03-Gal4 with either UAS-mCD8::GFP (for Draper antibody) or UAS-mCD8::mCherry (for Draper MiMIC) were dissected at 90 h after pupal formation (hAPF) at the stage when legs begin to twitch, and at 0, 3, and 12 DPE. For Draper detection via antibody, brains were incubated with either a combination of chicken anti-GFP (Aves Labs; 1:1,000), rabbit anti-GAT (gift from M. Freeman, OHSU; 1:2,000), and mouse anti-Draper (DSHB 8A1; 1:400) in conjunction with donkey anti-chicken 488 (Jackson 703-545-155; 1:100), donkey anti-rabbit Cy3 (Jackson 711-165-152; 1:100), and donkey anti-mouse Cy5 (Jackson 715-175-151; 1:100). For expression analysis in Draper MiMIC flies, endogenous GFP-tagged Draper was visualized without antibody amplification, and brains were incubated with only rabbit anti-GAT (1:2,000) followed by donkey anti-rabbit Cy3 (1:100).

### 2.3. Imaging and Imaris analysis

For ORN masking, brains were imaged on a Zeiss LSM800 Laser Scanning Confocal Microscope using a 100 × 1.4 NA Plan-Apochromat lens at an optical zoom of 0.5× with a step size of 0.23 μm. Acquisitions were centered on the glomerulus in question and upper and lower z levels set based on the presence of mCD8::GFP signal indicating ORN terminal arbor. Laser intensity and gain were adjusted, for each image, to fully use the dynamic range of the detector. To visualize glial processes across a coronal section of the antennal lobe through VC1, brains were imaged as above except with a 63 × 1.4 NA Plan-Apochromat lens at an optical zoom of 1.3× in Airyscan mode at a step size of 0.19 μm. For Draper expression, brains were imaged on a 3i Spinning Disk Confocal Microscope using a 63 × 1.4 NA Plan-Apochromat lens with a step size of 0.5 μm. Laser intensity and gain were kept constant between samples of same stain.

Imaris 9.9.1 (Oxford Instruments) was then used for analysis of ORN terminal arbor morphology and presynaptic content. First, the Surfaces function was used to precisely trace mCD8::GFP signal to create a 3D representation of ORN terminal arbors synapsing onto each glomerulus. With the mCD8::GFP channel selected, the bounds of a rectangular region of interest were modified to roughly encapsulate ORN terminal arbor signal. The function then calculated a preliminary contour line on each z slice with parameters set to background subtraction (local contrast) with an automated threshold, diameter of largest sphere which fits into the object set to 10 μm, smoothing selected, and surfaces detail set to 0.25 μm. This boundary was visually checked to ensure it faithfully traced ORN terminal arbor across all z slices and, if necessary, the automatic threshold was minimally adjusted.

After creation of the surface, the slicer function was used to manually demarcate and remove axon from the ORN terminal arbor. Additionally, the surface was checked against mCD8::GFP signal in both volume and slice views and any extra inclusions were removed with the slicer function. The volume, surface area, and sphericity of the resulting surface was then reported. To quantify the presynaptic content of ORN-defined glomeruli, Brp signal was masked by the corresponding ORN terminal arbor surface and deconvoluted in Imaris. Brp puncta were characterized using the Imaris Spots function with an estimated diameter of 0.4 μm (based on manual measurement of 50 random Brp puncta in control flies) and region growing enabled. Briefly, our Spots creation parameters were as follows: background subtraction enabled, no modeling of PSF-elongation, quality filter threshold of above 2,000, Spot regions from absolute intensity, and automated Spots region threshold with diameter from region border. We note that both ORN terminal arbor volume and presynaptic content (Brp puncta count) are sensitive to genetic background as the control values for VC1 and VM7 are sensitive to the specific glial drivers used. Appropriate controls were compared to *drpr* knockdown animals in all experiments.

To quantify glial volume within ORN-defined glomeruli, mCD8::RFP signal was masked by the corresponding ORN terminal arbor surface. Glial processes were characterized using the Imaris Surfaces function with parameters as follows: background subtraction (local contrast) with a threshold of 10,000, diameter of largest sphere which fits into the object set to 1 μm, smoothing deselected, processes selected greater than 20 voxels, and surfaces detail set to 0.25 μm. To quantify Brp-short puncta, the Imaris Spots function was used with identical settings as above for Brp with the following exceptions: (1) deconvolution was not performed, (2) before detection, a rectangular region of interest was modified to roughly encapsulate the terminal arbor cluster of Brp-short puncta, and (3) after detection, the terminal arbor cluster was checked for stray inclusions which were then removed. The volume of the resulting surface was then reported, normalized to ORN terminal arbor volume. All quantification was performed with the user blind to genotype. Draper quantification was performed by measuring mean intensity in FIJI using at least 5 astrocytes and 5 ensheathing glia per brain, averaged to provide a mean value per cell type for each brain. Measurements were restricted to the glial cell bodies, and corresponding cell type was identified by the presence (astrocytes) or absence (ensheathing glia) of GAT staining.

### 2.4. Statistical analysis

**FIGURE 1:**
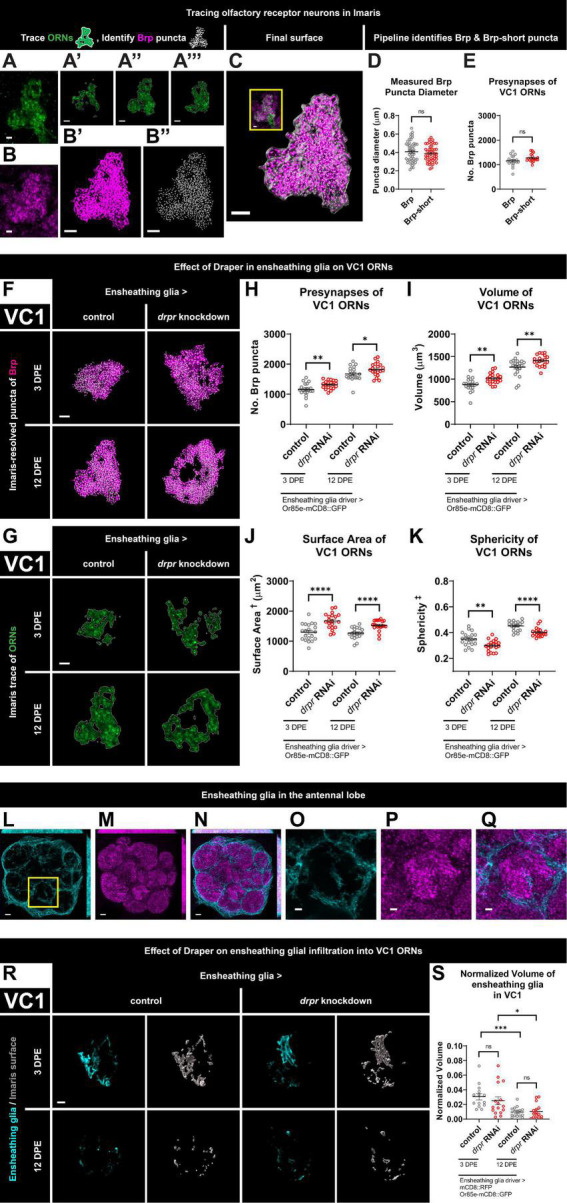
Loss of Draper in ensheathing glia leads to persistent increases in presynaptic content and terminal arbor size of VC1 ORNs. **(A)** Immunohistochemistry stain (single slice) followed by **(A’–A”’)** precise, semi-automated tracing (three sample slices shown) of pb2A ORN terminal arbor membrane in flies expressing Or85e-mCD8::GFP. **(B)** Brp staining (single slice), **(B’)** MIP of deconvoluted Brp staining, **(B”)** center points of recognized Brp puncta (MIP) inside pb2A ORN terminal arbors. **(C)** Surface of pb2A ORN terminal arbors recognized by Imaris (gray) with MIP of deconvoluted Brp channel (magenta) and recognized puncta (white) inside compared to original overlay of confocal channels (inset). **(D)** Both Brp and Brp-short puncta have a mean diameter of 0.4 μm (manual measurement). **(E)** Imaris pipeline detects comparable Brp and Brp-short puncta numbers in VC1 ORNs. **(F)** MIP overlays of Brp stain (magenta) and recognized puncta (white), **(G)** single coronal slices of membrane staining (green) and traces (white) of pb2A ORN terminal arbors in control flies and flies with constitutive knockdown of *drpr* in ensheathing glia at 3 DPE and 12 DPE. Draper in ensheathing glia persistently limits **(H)** presynapse number, **(I)** volume, **(J)** surface area, and **(K)** persistently promotes sphericity of VC1 ORN terminal arbors. In a coronal MIP through the antennal lobe (3.5 μm), **(L)** ensheathing glia processes (cyan), **(M)** Brp staining (magenta), **(N)** overlay. In the region (inset of L) surrounding VC1, **(O)** ensheathing glia processes (cyan), **(P)** Brp staining (magenta), **(Q)** overlay. **(R)** MIP of ensheathing glia processes (cyan) in the interior of VC1 ORN terminal arbors and recognized glial surface (gray) in control flies and flies with constitutive knockdown of *drpr* in ensheathing glia at 3 DPE and 12 DPE. **(S)** Volume of ensheathing glia inside VC1 ORN terminal arbors normalized to terminal arbor volume. Ensheathing glia are more infiltrative into VC1 at 3 DPE than 12 DPE. ^†^Surface area is calculated based on light-level confocal microscopy measurements. ^‡^Sphericity is the ratio of the surface area of an equal-volume sphere to the surface area of an object and ranges from 0 to 1 (most spherical). For each condition, *n* > 16 antennal lobes from 8 brains. *p < 0.05, **p < 0.01, ***p < 0.001, ****p < 0.0001, and ns = not significant. All error bars represent mean ± SEM. See (section 2.4. “Statistical analysis”) for details of statistical tests used. Genotypes: **(A,A’–A”’,B,B’,B”,C)** + /Or85e-mCD8::GFP; + /MZ0709-Gal4. **(D,E)** Brp is + /Or85e-mCD8::GFP; + /MZ0709-Gal4. Brp-short is Or85e-Gal4/UAS-Brp-short-mCherry; + /Dr & Or85e-Gal4/UAS-Brp-short-mCherry; + /TM3Sb pooled. **(F–K)** Control is + /Or85e-mCD8::GFP; + /MZ0709-Gal4. *drpr* knockdown & *drpr* RNAi is + /Or85e-mCD8::GFP; UAS-*drpr* RNAi/MZ0709-Gal4. **(L–Q)** + /UAS-mCD8::GFP; + /GMR5 6F03-Gal4. **(R,S)** Control is UAS-mCD8::RFP/Or85e-mCD8::GFP; UAS-Luciferase/MZ0709-Gal4. *drpr* knockdown & *drpr* RNAi is UAS-mCD8::RFP/Or85e-mCD8::GFP; UAS-*drpr* RNAi/MZ0709- Gal4. Scale bar = 2 μm (**A,B**, inset of **C,O–Q**), 3 μm **(A’–A”’,B’,B”,C,F,G,R)**, 5 μm **(L–N)**.

**FIGURE 2:**
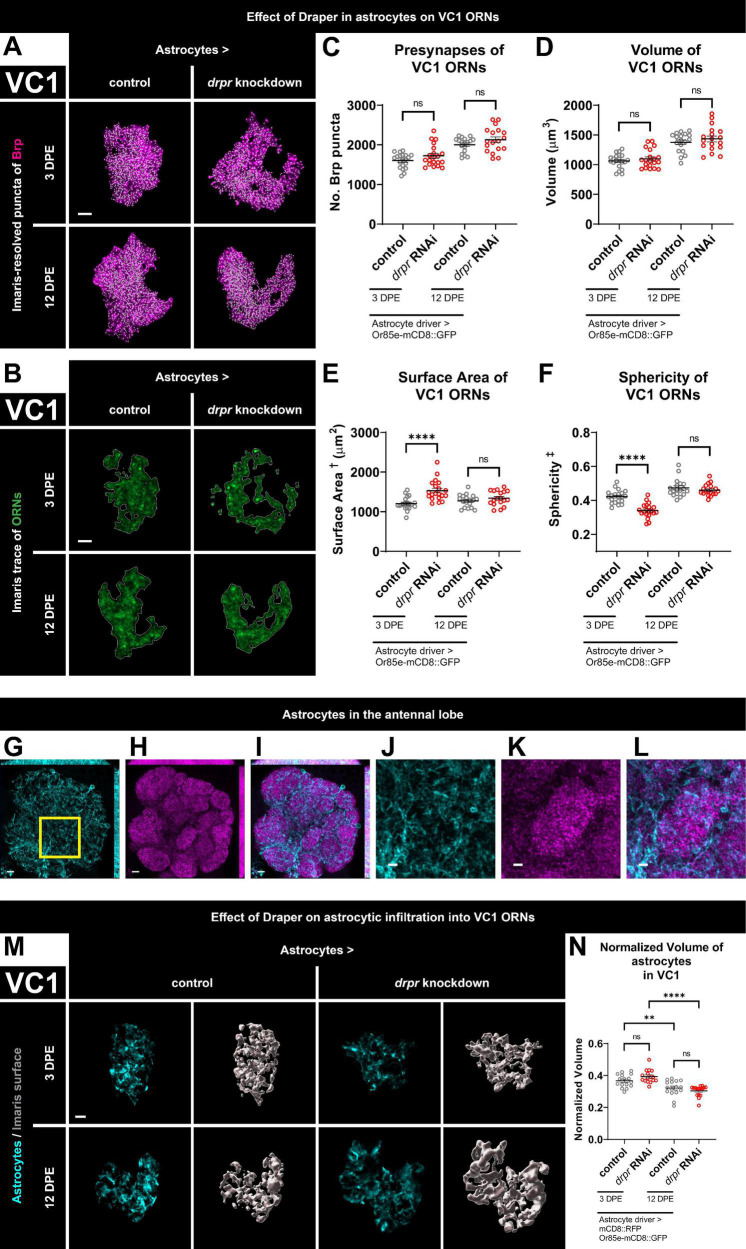
Loss of Draper in astrocytes does not alter VC1 ORN presynaptic content or terminal arbor size, but leads to transient changes in glomerular shape. **(A)** MIP overlays of Brp stain (magenta) and recognized puncta (white), **(B)** single coronal slices of membrane staining (green) and traces (white) of pb2A ORN terminal arbors in control flies and flies with constitutive knockdown of *drpr* in astrocytes at 3 DPE and 12 DPE. Draper in astrocytes does not affect **(C)** presynapse number or **(D)** volume but transiently limits **(E)** surface area and transiently promotes **(F)** sphericity of VC1 ORN terminal arbors. In a coronal MIP through the antennal lobe (3.5 μm), **(G)** astrocyte processes (cyan), **(H)** Brp staining (magenta), **(I)** overlay. In the region (inset of **G**) surrounding VC1, **(J)** astrocyte processes (cyan), **(K)** Brp staining (magenta), **(L)** overlay. **(M)** MIP of astrocyte processes (cyan) in the interior of VC1 ORN terminal arbors and recognized glial surface (gray) in control flies and flies with constitutive knockdown of *drpr* in astrocytes at 3 DPE and 12 DPE. **(N)** Volume of astrocytes inside VC1 ORN terminal arbors normalized to terminal arbor volume. Astrocytes are more infiltrative into VC1 at 3 DPE than 12 DPE. ^†^Surface area is calculated based on light-level confocal microscopy measurements. ^‡^Sphericity is the ratio of the surface area of an equal-volume sphere to the surface area of an object and ranges from 0 to 1 (most spherical). For each condition, *n* > 16 antennal lobes from 8 brains. **p < 0.01, ****p < 0.0001, and ns = not significant. All error bars represent mean ± SEM. See (section 2.4. “Statistical analysis”) for details of statistical tests used. Genotypes: **(A–F)** control is + /Or85e-mCD8::GFP; + /R86E01-Gal4. *drpr* knockdown & *drpr* RNAi is + /Or85e- mCD8::GFP; UAS-*drpr* RNAi/R86E01-Gal4. **(G–L)** + /UAS-mCD8::GFP; + /GMR25H07-Gal4. **(M,N)** Control is UAS-mCD8::RFP/Or85e-mCD8::GFP; UAS-Luciferase/R86E01-Gal4. *drpr* knockdown & *drpr* RNAi is UAS-mCD8::RFP/Or85e-mCD8::GFP; UAS-*drpr* RNAi/R86E01-Gal4. Scale bar = 2 μm **(J–L)**, 3 μm **(A,B,M)**, 5 μm **(G–I)**.

**FIGURE 3:**
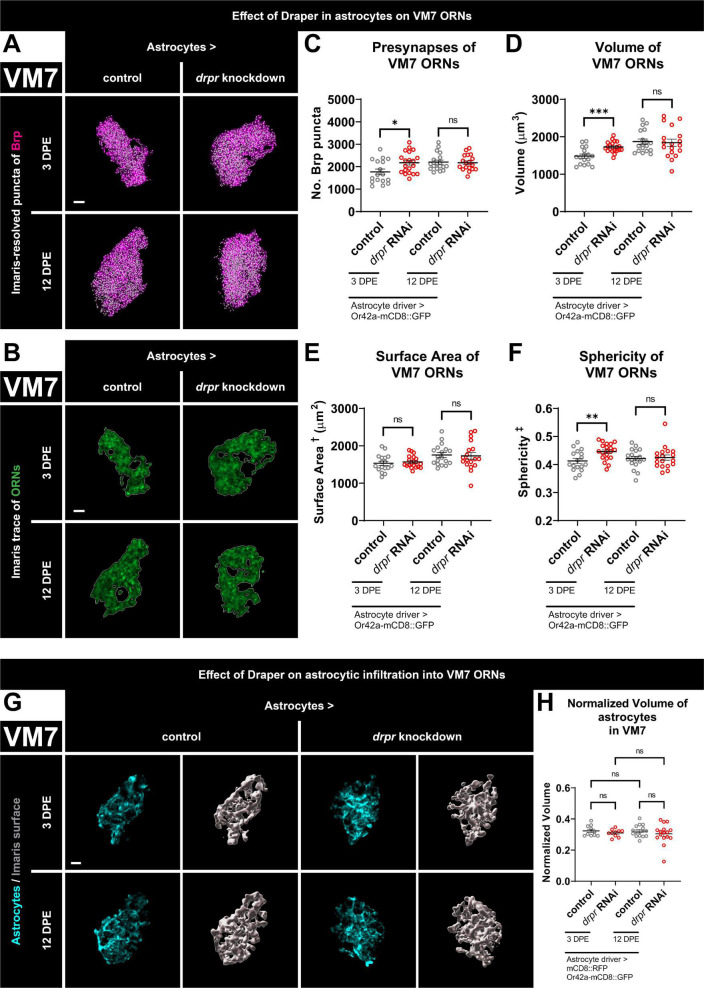
Loss of Draper in astrocytes results in transient increases in presynaptic content and terminal arbor size of VM7 ORNs. **(A)** MIP overlays of Brp stain (magenta) and recognized puncta (white), **(B)** single coronal slices of membrane staining (green) and traces (white) of pb1A ORN terminal arbors in control flies and flies with constitutive knockdown of *drpr* in astrocytes at 3 DPE and 12 DPE. Draper in astrocytes transiently limits **(C)** presynapse number, **(D)** volume, and **(F)** sphericity but not **(E)** surface area of VM7 ORN terminal arbors. **(G)** MIP of astrocyte processes (cyan) in the interior of VM7 ORN terminal arbors and recognized glial surface (gray) in control flies and flies with constitutive knockdown of *drpr* in astrocytes at 3 DPE and 12 DPE. **(H)** Volume of astrocytes inside VM7 ORN terminal arbors normalized to terminal arbor volume. Astrocytes are equally infiltrative into VM7 at 3 DPE and 12 DPE. ^†^Surface area is calculated based on light-level confocal microscopy measurements. ^‡^Sphericity is the ratio of the surface area of an equal-volume sphere to the surface area of an object and ranges from 0 to 1 (most spherical). For each condition, *n* > 16 antennal lobes from 8 brains. *p < 0.05, **p < 0.01, ***p < 0.001, and ns = not significant. All error bars represent mean ± SEM. See (section 2.4. “Statistical analysis”) for details of statistical tests used. Genotypes: **(A–F)** control is + /Or42a-mCD8::GFP; + /R86E01-Gal4. *drpr* knockdown & *drpr* RNAi is + /Or42a-mCD8::GFP; UAS-*drpr* RNAi/R86E01-Gal4. **(G,H)** Control is UAS-mCD8::RFP/Or42a-mCD8:GFP; UAS-Luciferase/R86E01-Gal4. *drpr* knockdown & *drpr* RNAi is UAS-mCD8::RFP/Or42a-mCD8:GFP; UAS-*drpr* RNAi/R86E01-Gal4. Scale bar = 3 μm.

All data were analyzed and graphed with Prism 9 (GraphPad Software). For the data in Figures 1D, E, H–K, 2C–F, 3C–F and Supplementary Figures 3A3–6, the Kolmogorov–Smirnov test for normality (α = 0.05) was performed on Brp-short & Brp puncta diameter, Brp-short & Brp puncta number, ORN terminal arbor volume, surface area, and sphericity for each genotype at each time point. Out of 68 datasets, 64 passed and the four that failed had *p*-values close to 0.05. Examination of the Q-Q plots for all 68 datasets, including the three that failed, revealed that quantile points lie close to the theoretical normal line, so normality is a safe assumption. For the two comparisons between Brp-short & Brp puncta diameter and between Brp-short & Brp puncta number, we used the *F* test (α = 0.05) to compare variances prior to choosing a statistical test. Both passed and we used an unpaired *t*-test (α = 0.05) to determine significance for these two comparisons. For the 32 comparisons between control and *drpr* RNAi genotypes, we used the *F* test (α = 0.05) to compare variances prior to choosing a statistical test. Out of 32 comparisons, two failed, but with *p*-values close to 0.05. We used an unpaired *t*-test with Welch’s correction (α = 0.05) to determine significance for these two comparisons and an unpaired *t*-test (α = 0.05) for the remaining 30.

To assess control genotype variability in Brp puncta number, ORN terminal arbor volume, surface area, and sphericity, we ran 16 additional comparisons between MZ0709 control and R86E01 control genotypes between Figures 1H–K, 2C–F; and between Figures 3C–F and Supplementary Figures 3A3–6. Normality was already established above. We used the *F* test (α = 0.05) to compare variances prior to choosing a statistical test. Out of 16 comparisons, one failed. We used an unpaired *t*-test with Welch’s correction (α = 0.05) to determine significance for this comparison and an unpaired *t*-test (α = 0.05) for the remaining 15. For VC1 ORN terminal arbors at 3 DPE, we note that flies expressing the R86E01-Gal4 driver show no significant difference in surface area from MZ0709-Gal4 expressing flies but are larger, more spherical, and have more Brp puncta. At 12 DPE, these genotypic differences do not persist for volume and sphericity but remain for presynaptic content. For VM7 ORN terminal arbors at 3 DPE, we note that flies expressing the R86E01-Gal4 driver show no significant differences in volume, surface area, or sphericity from MZ0709-Gal4 expressing flies but have less Brp puncta. At 12 DPE, this genotypic difference in presynaptic content does not persist; R86E01-Gal4 flies have a slight increase in sphericity.

For the data in Figures 1S, 2N, 3H and Supplementary Figure 3A8, the Kolmogorov–Smirnov test for normality (α = 0.05) was performed on normalized glial volume inside the glomerulus. Of 16 datasets, 13 passed and 3 failed. Examination of the Q-Q plots revealed that quantile points did lie close to the theoretical normal line for the 13 datasets that passed as well as one that failed but deviated from normality for two that failed. Thus, normality is a safe assumption for 14 of 16 datasets. For the 12 comparisons involving datasets that are normally distributed, we used the *F* test (α = 0.05) to compare variances prior to choosing a statistical test. Out of 12 comparisons, one failed. We used an unpaired *t*-test with Welch’s correction (α = 0.05) to determine significance for this comparison and an unpaired *t*-test (α = 0.05) for the remaining 11. For the 4 comparisons involving datasets that are not normally distributed, we used a Mann–Whitney test (α = 0.05) to determine significance.

**FIGURE 4:**
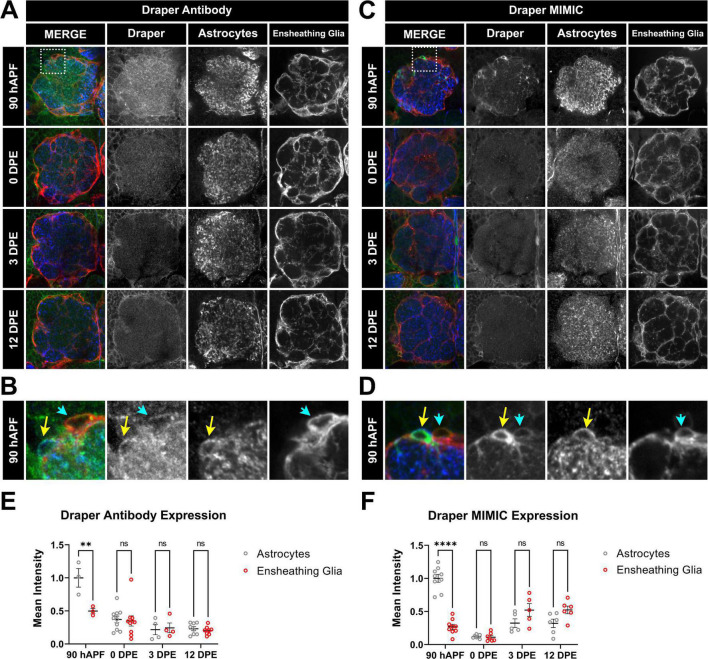
Time course of Draper expression in antennal lobe ensheathing glia and astrocytes. **(A–D)** Draper expression was visualized at 90 hAPF, 0 DPE, 3 DPE, and 12 DPE with either rabbit anti-Draper antibody **(A,B)** or a protein trap cassette insertion containing EGFP that allows for endogenous Draper protein visualization **(C,D)**. Insets from 90 hAPF images depict relative Draper expression in adjacent astrocytes and ensheathing glial cells **(B,D)**. **(E,F)** Quantification of Draper expression in cell bodies of astrocytes (gray circles) and ensheathing glia (red circles) at 90 hAPF, 0 DPE, 3 DPE, and 12 DPE, normalized to average intensity of astrocytes at 90 hAPF. Data shown as mean intensity per animal from at least 5 cells of each glial subtype. **p < 0.01, ****p < 0.001, and ns = not significant. All error bars represent mean ± SEM. See (section 2.4. “Statistical analysis”) for details of statistical tests used. Genotypes: **(A,B)** w;GMR56F03-Gal4, UAS-mCD8:GFP/ + with ensheathing glia (anti-GFP pseudocolored red), Draper (anti-Draper antibody, green), and astrocytes (anti-GAT antibody, blue). **(C,D)** w;GMR56F03-Gal4,UAS-mCD8:mCherry/Mi{PT-GFSTF.1}drpr[MI07659-GFSTF.1] to visualize endogenous Draper-EGFP (green), endogenous mCherry for ensheathing glia (red), and anti-GAT for astrocytes (blue).

For the data in Figure 4, we used a two-way ANOVA with Šídák’s multiple comparisons test (α = 0.05) to determine significance between mean intensity of Draper signal within astrocytes and ensheathing glia cell bodies at different time points. For the data in Supplementary Figure 1B, the Kolmogorov–Smirnov test for normality (α = 0.05) was performed on Brp puncta diameter for each genotype at each time point. Out of 16 datasets, 12 passed and four failed. Examination of the Q-Q plots revealed that quantile points did lie close to the theoretical normal line for the 12 datasets that passed but deviated from normality for the four that failed. For the 12 normally distributed datasets, we used the *F* test (α = 0.05) to confirm that, for each of these six comparisons, the variances were not significantly different. We used an unpaired *t*-test (α = 0.05) to determine significance for these six comparisons. We used a Mann–Whitney test (α = 0.05) to determine significance for the remaining two comparisons on the four datasets that failed normality.

## 3. Results

### 3.1. Quantification of ORN architecture and presynaptic content in Imaris

To quantify ORN morphology and presynaptic content, we developed a pipeline to precisely mask mCD8::GFP-labeled ORN terminal arbors using the Surfaces function in Imaris and then quantified Brp puncta specifically within the ORN mask. Brp is a structural component of the presynaptic T-bar with homology to mammalian ELKS/CAST/ERC (Wagh et al., 2006) and is widely used as a presynaptic marker in Drosophila. The mask was also employed to quantify glial infiltration within glomeruli (see below). Thus, we obtained readouts of ORN terminal arbor morphology, presynaptic content, and glial infiltration. We chose to quantify endogenous Brp by masking defined ORN terminal arbors as it avoids Gal4-dependent overexpression in UAS-driven tagged Brp-short (Duhart and Mosca, 2022), and simplifies genetic analyses of glial requirements in circuit development. This protocol was inspired by prior work in the field (Mosca and Luo, 2014; Mosca et al., 2017; Aimino et al., 2023).

We briefly describe the process of creating the mask for the VC1 glomerulus, but the procedure is the same for VM7. First, brains are imaged on a confocal microscope with upper and lower z levels set based on presence of the ORN terminal arbor a coronal slice through VC1 is shown in Figures 1A, B. After importing the z stack into Imaris, a rectangular region of interest is modified to encompass the ORN terminal arbor. The Surfaces function then calculates contour lines on each z slice (Figures 1A’–A”’), which are manually checked against the membrane stain. After the surface is created, the slicer tool is used to remove axon and any unwanted inclusions. This surface is comprised of the packed axon terminals of VC1 ORNs. Then, this surface is used to mask Brp signal, the signal is deconvoluted (Figure 1B’), and the Spots function detects Brp puncta (Figure 1B”) inside the final ORN terminal arbor surface (Figure 1C). More detailed illustration of this pipeline is available (Supplementary Figure 1A). By super-resolution approaches, Brp forms ring structures with a diameter of roughly 200 nm (Kittel et al., 2006; Hoover et al., 2019). However, visualized with standard confocal microscopy, Brp puncta appear as diffraction limited spots, here with a mean diameter of 0.4 μm (Figure 1D). This matches the diameter of Brp-short puncta (Figure 1D), and puncta size was consistent across genotypes (Supplementary Figure 1B). Importantly, VC1 Brp puncta number calculated using this method matches estimation of presynaptic content via Brp-short expressed specifically in VC1 ORNs (Figure 1E). Thus, quantifying endogenous Brp puncta within masked ORN terminal arbors is comparable to cell-type specific overexpression of tagged Brp-short. In conclusion, our pipeline provides a high-throughput method to mask ORN terminal arbors and enables screening for genes required for ORN architecture, regulation of presynaptic protein levels of any protein with available antibodies, as well as glial infiltration.

We assessed ORN terminal arbor volume and presynaptic content at both 3 DPE and 12 DPE to assess the timing of Draper-dependent effects. It was recently shown that ORN terminal arbor size and Brp puncta increase markedly in multiple glomeruli across early adulthood (Aimino et al., 2023). Thus, we assessed these metrics in control and *drpr* knockdown animals at 3 DPE to assess if Draper-dependent synapse refinement is apparent during a period of net neuropil growth. By 12 DPE, ORN terminal arbor volume and presynaptic content have stabilized (Aimino et al., 2023). Thus, we chose this later time point to test requirements for glial *drpr* well after the phase of synapse addition in the antennal lobe is complete.

### 3.2. Loss of Draper in ensheathing glia leads to persistent increases in presynaptic content and terminal arbor size of VC1 ORNs

We first examined changes to VC1 ORN terminal arbor presynapse number and morphology at 3 DPE and 12 DPE following knockdown of *drpr* in ensheathing glia (Figures 1F–K and Supplementary Figures 1C, D). At 3 DPE, *drpr* knockdown animals have a 14% increase in presynaptic content relative to age-matched controls (Figures 1F, H and Supplementary Figure 1D). Mirroring the increase in Brp puncta, VC1 ORNs exhibit a 16% increase in VC1 ORN terminal arbor volume (Figures 1F, I and Supplementary Figures 1C, D). To comprehensively characterize the morphology of ORN terminals, we sought relevant metrics for shape. We propose ORN terminal arbor surface area and sphericity as two metrics for analyzing antennal lobe organization and compartmentalization. Sphericity is defined as the ratio of the surface area of an equal-volume sphere to the actual surface area of an object (Wadell, 1935). Thus, for a given volume, neuron terminal arbors with a higher surface area are predicted to have lower sphericity. While confocal-based approaches to resolve precise ORN terminal arbor borders will affect absolute values for volume, surface area, and sphericity, this limitation applies equally to each genotype. Compared to 3 DPE control animals, *drpr* knockdown flies exhibit a 29% increase in VC1 terminal arbor surface area (Figures 1G, J and Supplementary Figures 1C, D) and a 15% decrease in sphericity (Figures 1G, K and Supplementary Figures 1C, D). Within controls, we note that gross glomerular shape is non-uniform. The disorganized shape of VC1 ORNs in *drpr* knockdown animals leads to many terminals having a doughnut appearance, instead of a round shape. The relatively dramatic effects of *drpr* knockdown on ORN surface area were unexpected and argue that Draper is required for proper organization of synaptic terminals in the antennal lobe. Together, these data demonstrate that loss of Draper in ensheathing glia results in modest increases in presynaptic content and ORN terminal arbor volume, as well as more pronounced changes in terminal arbor architecture at 3 DPE.

We next assessed the consequences of *drpr* knockdown in ensheathing glia in VC1 ORNs at 12 DPE. Flies with *drpr* knockdown have 9% more Brp puncta and an 11% increase in ORN terminal arbor volume relative to age-matched controls (Figures 1F, H, I and Supplementary Figures 1C, D). In addition, VC1 ORN terminals from *drpr* knockdown animals display a 27% increase in surface area and an 11% decrease in sphericity (Figures 1G, J, K and Supplementary Figures 1C, D). Together, these data indicate that Draper is required in ensheathing glia to limit presynapse number, volume, and surface area of VC1 ORN terminal arbors. These Draper-dependent phenotypes are comparable between early and mid-adulthood. Importantly, because loss of Draper results in phenotypes at 3 DPE, when presynaptic content and antennal lobe size are still increasing (Figures 1H, I), Draper-mediated signaling in ensheathing glia restrains VC1 ORN presynaptic size before the terminals reach their full size.

Because ensheathing glia are best known to wrap individual glomeruli (Freeman, 2015; Wu et al., 2017), but not thought to infiltrate into synapse-rich regions in the absence of injury, we investigated whether their processes are present in the glomerular interior of ORNs. As expected, ensheathing glial membranes labeled with mCD8::RFP (pseudocolored cyan) are predominantly found surrounding individual glomeruli (Figures 1L–Q). However, when we focus on those processes within individual glomeruli by first applying the ORN mask, we observe sparse ensheathing glia processes within the VC1 ORN terminal mask at both timepoints (Figure 1R). Ensheathing glia are not observed to wrap glomeruli in these visualizations (Figure 1R) because the mask covers only the ORN terminal volume within the glomeruli, not the surrounding region where the majority of ensheathing glial processes are found (Figures 1L, O). Interestingly, infiltrating processes are more substantial (normalized to VC1 glomerular volume) in early adulthood (Figure 1S). We next tested if loss of Draper from ensheathing glia changes their capacity for infiltration. We do not detect a change in ensheathing glial infiltration volume in *drpr* knockdown animals at either time point (Figure 1S), indicating that Draper does not regulate ensheathing glial infiltration into VC1 ORNs.

### 3.3. Loss of Draper in astrocytes does not alter VC1 ORN presynaptic content or terminal arbor size, but leads to transient changes in glomerular shape

Having established that Draper in ensheathing glia regulates VC1 ORN terminal arbor size and presynaptic content, we investigated if there is any requirement for astrocytic Draper, as it is required for the dramatic synaptic clearance that occurs throughout the CNS in early metamorphosis (Tasdemir-Yilmaz and Freeman, 2014). As before, we quantified Brp puncta and terminal arbor volume. At 3 DPE, we did not observe changes in either presynaptic content or ORN terminal arbor volume following knockdown of Draper in astrocytes (Figures 2A, C, D and Supplementary Figures 2A, B). Surprisingly, although astrocytic Draper does not regulate the size of VC1 ORN terminal arbors, it does regulate their shape. Animals with loss of Draper in astrocytes display a 27% increase in surface area and a 19% decrease in sphericity at 3 DPE (Figures 2B, E, F and Supplementary Figures 2A, B). Thus, Draper-mediated signaling in astrocytes regulates VC1 ORN terminal architecture, independent of overall size and presynaptic content at 3 DPE.

To test for a requirement for Draper in astrocytes later in adulthood, we investigated whether VC1 phenotypes are observed at 12 DPE. However, at this time point, we do not observe changes in Brp puncta, ORN terminal arbor size or shape (Figures 2A–F and Supplementary Figures 2A, B). In summary, Draper in astrocytes is required for proper VC1 terminal arbor shape at 3 DPE, though this effect is transient and is no longer observed at 12 DPE. We do not observe a role for astrocytic Draper in setting arbor size or presynaptic content at either time point. Taken together, these data indicate that for VC1, ensheathing glial Draper plays a more prominent role than astrocytic Draper in regulating ORN presynaptic terminals.

We investigated whether astrocytic infiltration into VC1 ORN terminals depends either on age or on Draper by labeling astrocytic membranes with mCD8::RFP and quantifying astrocytic volume specifically within the ORN terminal arbor mask. As expected, we find extensive astrocytic infiltration at both 3 DPE and 12 DPE (Figures 2G–M). Interestingly, astrocytes are more extensively infiltrated into VC1 ORN terminals in early adulthood (Figure 2N), consistent with the time course of ensheathing glia infiltration (Figure 1S), suggesting that both glial subtypes may play age-dependent functions in VC1. On the other hand, we do not find a requirement for Draper in the regulation of astrocytic infiltration into VC1 ORNs (Figure 2N).

### 3.4. Loss of Draper in ensheathing glia does not alter presynaptic content, glomerular size, or shape of VM7 ORNs

We were curious if the findings we observed for VC1 applied generally to all antennal lobe glomeruli. VM7 ORNs, responsible for detecting food volatiles, were of particular interest since they exhibit striking early life plasticity. Exposure to ethyl butyrate from 0 to 2 DPE leads to a reduction in volume of VM7 ORN presynaptic terminals, while ethyl butyrate exposure later in life does not (Golovin et al., 2019). Thus, we assessed phenotypic consequences of loss of glial Draper both immediately after an early life critical period (3 DPE), and when these terminals exhibit reduced activity-dependent plasticity (12 DPE).

Representative maximum intensity projections for Brp puncta in corresponding arbor interiors (Supplementary Figure 3A1), membrane tracing of a selected coronal slice through ORNs (Supplementary Figure 3A2), and ORN terminal arbor surfaces (Supplementary Figure 3B) are shown at 3 DPE and 12 DPE for controls and animals with *drpr* knockdown. Surprisingly, we find that knockdown of *drpr* in ensheathing glia does not affect VM7 ORN presynapse number, volume, surface area, or sphericity at either time point (Supplementary Figures 3A3–6). Moreover, we do not detect Draper-dependent changes in ensheathing glial infiltration into VM7 ORNs (Supplementary Figures 3A7, 8). Thus, while Draper signaling in ensheathing glia regulates presynaptic characteristics of VC1 ORNs, we do not detect a function for ensheathing glial Draper in regulating size, morphology, or presynaptic content of VM7 ORNs.

### 3.5. Loss of Draper in astrocytes results in transient increases in presynaptic content and terminal arbor size of VM7 ORNs

The apparent lack of regulation of VM7 ORNs by ensheathing glial Draper suggested either that Draper-dependent signaling does not refine these terminals or that they depend on Draper signaling in a different cell type. To differentiate between these possibilities, we tested for a Draper requirement in astrocytes. We present representative maximum intensity projections of masked Brp puncta (Figure 3A), single coronal slices of ORN terminal arbor membrane (Figure 3B), recognized surfaces (Supplementary Figure 4A), and videos including recognized Brp puncta inside the final ORN surface (Supplementary Figure 4B) for controls and animals with *drpr* knockdown in astrocytes at both time points. At 3 DPE, we see a relatively large 23% increase in presynaptic content and a 16% increase in ORN terminal arbor volume in *drpr* astrocyte knockdown flies relative to controls (Figures 3C, D). However, the shape of VM7 ORN terminal arbors is not regulated by astrocytic Draper since we do not detect a change in surface area in these animals (Figure 3E). This contrasts the role that ensheathing glial Draper plays in regulating the overall architecture of VC1 (Figure 1). We do see a modest 8% increase in ORN sphericity in these *drpr* knockdown flies relative to controls (Figure 3F), which is expected given the increase in volume without a corresponding increase in surface area. Surprisingly, these phenotypes are transient and do not persist until 12 DPE (Figures 3A–F and Supplementary Figures 4A, B). Consistent with a role for Draper-mediated signaling in astrocytes shaping VM7 ORN terminal arbor volume and presynaptic content, we observe extensive astrocytic infiltration in this glomerulus at both time points (Figure 3G). However, we do not see a change in normalized infiltration volume between time points or following *drpr* knockdown (Figure 3H).

In summary, these data indicate that astrocytic Draper limits presynaptic content and terminal arbor volume of VM7 ORNs. The fact that these phenotypes are observed at 3 DPE indicates that Draper-dependent refinement of VM7 circuitry is occurring by the end of the proposed critical period for this glomerulus (Golovin et al., 2019). However, the consequences of loss of astrocytic Draper are not observed at 12 DPE, arguing that given enough time, alternative mechanisms compensate for the lack of astrocytic Draper in VM7 (see section “4. Discussion”).

### 3.6. Draper expression peaks before adulthood in antennal lobe astrocytes

In the above experiments, we knocked down *drpr* constitutively from embryonic stages until early and mid-adulthood to examine cumulative changes to ORN terminal arbor presynaptic content, size, and shape. In VC1, we uncovered a persistent role for ensheathing glial Draper in limiting ORN terminal arbor presynaptic content, size, and shape; and a transient role for astrocytic Draper in setting ORN terminal arbor shape. In VM7, astrocytic Draper plays a transient role in limiting ORN terminal arbor presynaptic content and size at 3 DPE, but the phenotype is no longer observed at 12 DPE. We were interested whether Draper levels in astrocytes and ensheathing glia parallel these findings.

Using two different methods to visualize Draper expression including an endogenous Draper MiMIC approach, whereby genomic Draper is marked with an EGFP-FlAsH-StrepII-TEV-3xFlag (GFSTF) tag to allow for visualization of endogenous protein expression (Nagarkar-Jaiswal et al., 2015), in parallel with an anti-Draper antibody stain, we quantified Draper levels in these glial populations across the antennal lobe both before and throughout early adulthood (Figure 4). Although the Draper MIMIC line shows more cytoplasmic expression, the two methods depict expression in the same cells. By both approaches, we observe high levels of Draper in astrocytes at late pupal stages 90 hAPF (yellow arrow in Figures 4B, D) which drop sharply into adulthood (Figures 4E, F). This unexpected peak of Draper in astrocytes in late pupal stage suggests that pruning occurs contemporaneously with synapse formation and circuit assembly in the antennal lobe (Muthukumar et al., 2014). Moreover, strong Draper expression in pupal astrocytes is consistent with the synaptic phenotypes observed at 3 DPE in both VC1 and VM7 ORNs (Figures 2, 3). By 12 DPE, compensation from other glia in the context of constitutive *drpr* knockdown in astrocytes may reverse astrocytic phenotypes. Ensheathing glia are known to express Draper constitutively and upregulate levels in response to injury (Doherty et al., 2009). Here we find that, in contrast to astrocytes, in ensheathing glia, there is no clear peak of Draper expression (Figures 4A, C, E, F). This is consistent with the all-or-nothing temporal ORN phenotypes we see on Draper knockdown in ensheathing glia. Taken together, Draper expression in both astrocytes and ensheathing glia is important to set up adult antennal lobe circuitry.

## 4. Discussion

Like the mammalian olfactory bulb, the Drosophila antennal lobe is segregated into well-defined synaptic territories called glomeruli. We set out to test if we could leverage its highly stereotyped anatomy to uncover molecular mechanisms regulating glial-dependent regulation of synaptic connectivity. As proof of concept, here we tested if the engulfment receptor Draper refines antennal lobe circuitry, since it is essential for glial-mediated clearance in the Drosophila CNS (Marin et al., 2005; Doherty et al., 2009; Tasdemir-Yilmaz and Freeman, 2014; McLaughlin et al., 2019). Specifically, we tested whether astrocytes and/or ensheathing glia require Draper to regulate presynaptic ORN terminals in two adult glomeruli: VC1 and VM7. Consistent with our hypothesis, we find that glial Draper limits overall volume and presynaptic content of ORN terminals in these two glomeruli. Surprisingly, we find glial subtype-specific requirements for Draper. While ensheathing glial Draper regulates the size and shape of VC1 ORNs, astrocytic Draper regulates VM7 ORNs. We probed VC1 and VM7 ORN morphology at both 3 DPE and 12 DPE and hypothesized that Draper-mediated refinement would be apparent at 12 DPE after the antennal lobe has reached its full size (Aimino et al., 2023). However, contrary to our expectations, we see evidence of Draper-mediated early life refinement of ORN presynaptic terminals while they are still in a phase of net growth. In line with an early role for Draper in restraining ORN volume and presynaptic content, it is strongly expressed in late pupal stage astrocytes and at relatively constant levels in ensheathing glia during both pupal and adult stages.

We show that loss of *drpr* in ensheathing glia increases the number of Brp puncta in VC1 ORNs at 3 DPE and 12 DPE whereas loss of *drpr* in astrocytes increases Brp puncta count in VM7 ORNs only at 3 DPE. Active zone number has been shown to be an accurate proxy for synapse number in the adult Drosophila antennal lobe (Mosca and Luo, 2014; Mosca et al., 2017; Aimino et al., 2023). In this work, we chose to quantify presynaptic puncta by volumetric mask of an antibody stain for Brp instead of other strategies (e.g., overexpression of tagged Brp-short) to obtain a readout of physiologic Brp expression devoid of Gal4-dependent overexpression or presence of an epitope tag (Duhart and Mosca, 2022). In addition, establishing a method for quantifying synaptic content based on endogenous protein expression simplifies the genetics required to probe glial pathways involved in synaptic refinement.

Our work demonstrates that both VC1 and VM7 increase in size over early adult life, consistent with volumetric expansion reported for other glomeruli over adulthood (Aimino et al., 2023). Interestingly, we find clear evidence that glial dependent synaptic refinement is occurring during a phase of net neuropil growth. Thus, synapse addition and synapse refinement are not temporally segregated in this circuit. We observe phenotypes at 3 DPE but do not know whether these phenotypes reflect Draper function during pupal development when the circuitry is just starting to set up or during early adulthood as an ongoing refinement process. Indeed, our findings that Draper is enriched in astrocytes during late pupal stages and drops into adulthood suggests the former. To our knowledge, this is the first reporting of Draper enrichment in antennal lobe astrocytes; however, these data fit with a previous report of increased Draper expression in late-stage pupal astrocytes in the optic lobe (Nakano et al., 2019). To determine the precise timing of glial-mediated circuit refinement within the antennal lobe, it will be important in future studies to temporally limit RNAi-mediated Draper knockdown (e.g., via Gal80^ts^) to refine Draper’s temporal requirement. It will be particularly interesting to test whether Draper signaling is required at the same time in ensheathing glia and astrocytes for refinement of antennal lobe circuitry. For example, Draper might act in astrocytes during pupal development and in ensheathing glia in adults, which might explain the early, transient phenotypes observed in VM7 with astrocyte knockdown.

Although Draper signaling in ensheathing glia and astrocytes limits terminal arbor volume and presynaptic content of VC1 and VM7 ORNs, respectively, interesting differences exist between its requirements in these two glomeruli. First, while ensheathing glial Draper limits the surface area of VC1, astrocytic Draper does not limit the surface area of VM7. In fact, Draper signaling in both ensheathing glia and astrocytes promotes sphericity of VC1, while we do not detect a requirement for Draper in either glial population for regulation of VM7 sphericity. Thus, glomerular architecture is regulated by glial Draper signaling in some, but perhaps not all, glomeruli. Second, while loss of Draper in ensheathing glia results in increased presynaptic content and terminal arbor volume of VC1 ORNs at both 3 DPE and 12 DPE, loss of Draper in astrocytes alters VM7 ORN presynaptic terminals only at 3 DPE.

Why does loss of Draper in astrocytes have only transient effects on VM7? We propose that in these animals, loss of astrocytic Draper is compensated for by either Draper function in another cell type, or activity of a parallel pathway. For example, it is possible that Draper signaling in ensheathing glia compensates for loss of Draper in astrocytes with respect to VM7 ORNs. To test this possibility, it will be important to investigate the effects of pan-glial Draper knockdown on presynaptic content, terminal arbor size, and morphology in VM7 ORNs. Alternatively, non-Draper-mediated refinement could be responsible for the phenotypic rescue observed at 12 DPE in VM7 ORNs. Of note, a second parallel pathway important for glial engulfment signals through the Crk/Mbc/dCed-12 guanine nucleotide exchange factor complex (Ziegenfuss et al., 2012). At metamorphosis onset when astrocytes transform into phagocytes, Draper and Crk/Mbc/dCed-12 act in parallel to clear Brp puncta from the CNS (Tasdemir-Yilmaz and Freeman, 2014).

Perhaps the most well-known example of stereotyped neurite pruning in Drosophila is of mushroom body γ neurites, mediated by astrocytes during metamorphosis (Marin et al., 2005; Tasdemir-Yilmaz and Freeman, 2014). From a morphological standpoint, with processes that infiltrate into the synaptic neuropil of individual glomeruli, astrocytes are well-poised to regulate synapse formation, function, and refinement (Freeman and Doherty, 2006; Muthukumar et al., 2014; Freeman, 2015; Kremer et al., 2017). Post-metamorphosis, astrocytes play key pro-synaptogenic roles. For example, in the antennal lobe, astrocyte ablation results in net synapse loss (Muthukumar et al., 2014). The data presented here open the door to astrocytes retaining Draper-dependent functions in refinement following CNS breakdown and into reconstruction. What is the functional consequence of loss of Draper signaling on antennal lobe circuit function? In mammalian models, loss of the Draper homolog MEGF10 in astrocytes increases excitatory synapse number and doubles spontaneous EPSC and miniature EPSC frequency in adult CA1 slices (Lee et al., 2021). Moving forward, it will be critical to test the functional effects of loss of Draper in distinct glial populations on ORN-projection neuron synaptic function. The Draper-mediated refinement model of the olfactory circuitry defined within this work, combined with powerful molecular tools that allow for widescale genetic manipulation, opens up exciting possibilities to investigate and define new cellular mechanisms of synaptic pruning and refinement in the CNS.

## Data availability statement

The raw data supporting the conclusions of this article will be made available by the authors, without undue reservation.

## Ethics statement

Ethical review and approval was not required for this study on *Drosophila melanogaster* in accordance with local legislation and institutional requirements.

## Author contributions

HB and JC-B obtained the funding and supervised the study. HB, JC-B, and DJ contributed to the conception and design of the study. HB and DJ wrote the original draft of the manuscript. HB, DJ, JC-B, and HL revised the manuscript. DJ, GS, and ES created the fly lines, performed the animal husbandry, dissected the adult brains, and performed the immunohistochemistry staining. DJ, JC-B, HL, and AF performed the confocal imaging. DJ and HL designed the Imaris analysis pipeline. DJ, HL, AW, ES, and JC-B executed image analysis. DJ, HL, and JC-B performed the statistical analysis. All authors contributed to the article and approved the submitted version.
