# Supplementary material for: Early Draper-mediated glial refinement of neuropil architecture and synapse number in the Drosophila antennal lobe
Source: Front Cell Neurosci. 2023 Jun 2;17:1166199. doi: 10.3389/fncel.2023.1166199 (PMC10272751; doi:10.3389/fncel.2023.1166199)
Supplement: Supplementary file 5 [file Data_Sheet_1.pdf]

Supplementary Material

See “Video 1.MP4”

Supplementary Figure 1A. (“Video 1.MP4”) Further illustration of Imaris pipeline used to mask ORNs and recognize Brp puncta. **(Upper left)** Step 1 - Imaris mask of ORNs (white line) using raw Or85e-mCD8::GFP signal (green). **(Upper right)** Step 2 - ORN mask (white line) overlayed with raw Brp signal (magenta). **(Bottom left)** Step 3 - Deconvoluted Brp signal (white) inside ORN mask (white line) overlayed with raw Brp signal (magenta). **(Bottom right)** Step 4 - Imaris-resolved Brp puncta (red) overlayed with deconvoluted Brp signal (white) inside ORN mask and raw Brp signal (magenta).

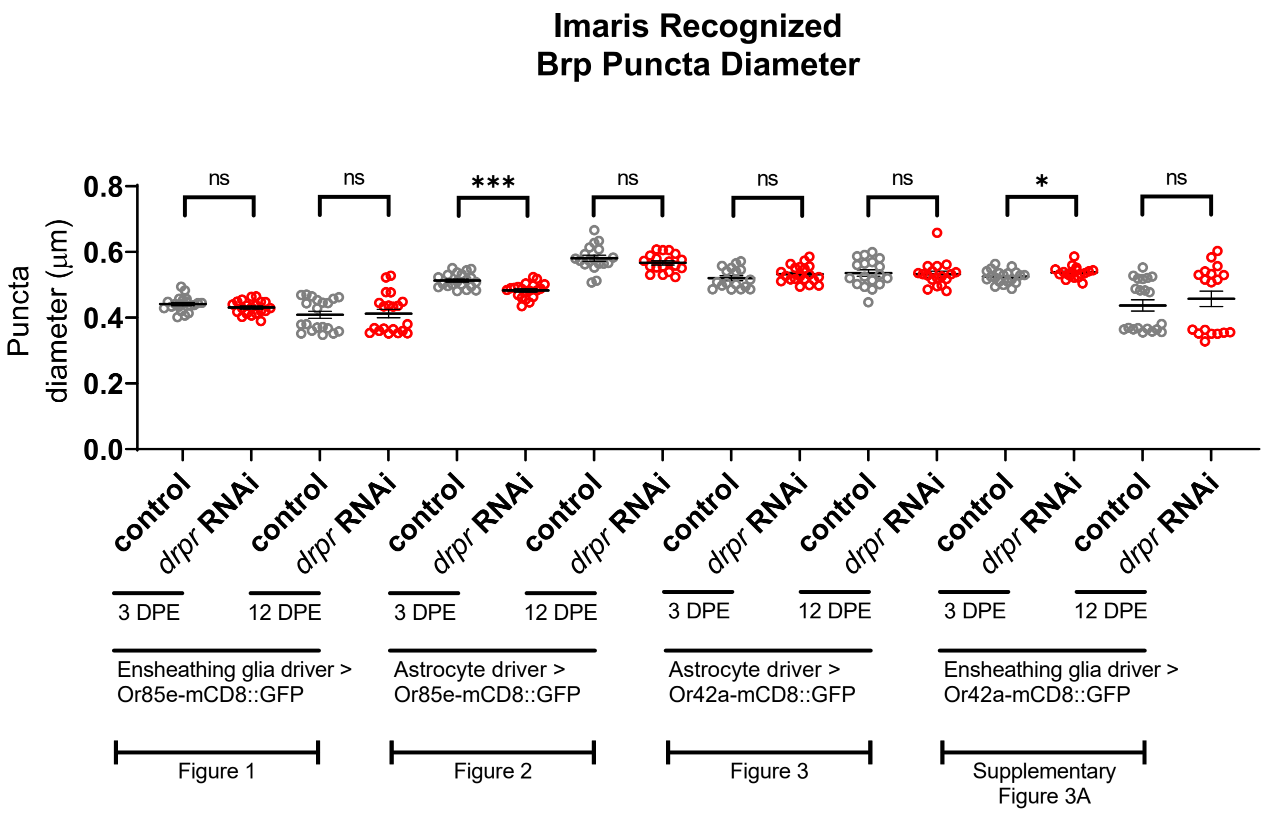

Supplementary Figure 1B. Imaris recognized Brp puncta diameter is mostly static across genotypes. For each condition, n > 16 antennal lobes from 8 brains. *$p < 0.05$, ***$p < 0.001$, and ns = not significant. All error bars represent means ± SEM. See (section 2.4. "Statistical analysis") for details of statistical tests used. Genotypes: **(Figure 1)** control is +/Or85e-mCD8::GFP; +/MZ0709-Gal4. *drpr* RNAi is +/Or85e-mCD8::GFP; UAS-*drpr* RNAi/MZ0709-Gal4. **(Figure 2)** control is +/Or85e-mCD8::GFP; +/R86E01-Gal4. *drpr* RNAi is +/Or85e-mCD8::GFP; UAS-*drpr* RNAi/R86E01-Gal4. **(Figure 3)** control is +/Or42a-mCD8::GFP; +/R86E01-Gal4. *drpr* RNAi is +/Or42a-mCD8::GFP; UAS-*drpr* RNAi/R86E01-Gal4. **(Supplementary Figure 3A)** control is +/Or42a-mCD8::GFP; +/MZ0709-Gal4. *drpr* RNAi is +/Or42a-mCD8::GFP; UAS-*drpr* RNAi/MZ0709-Gal4.

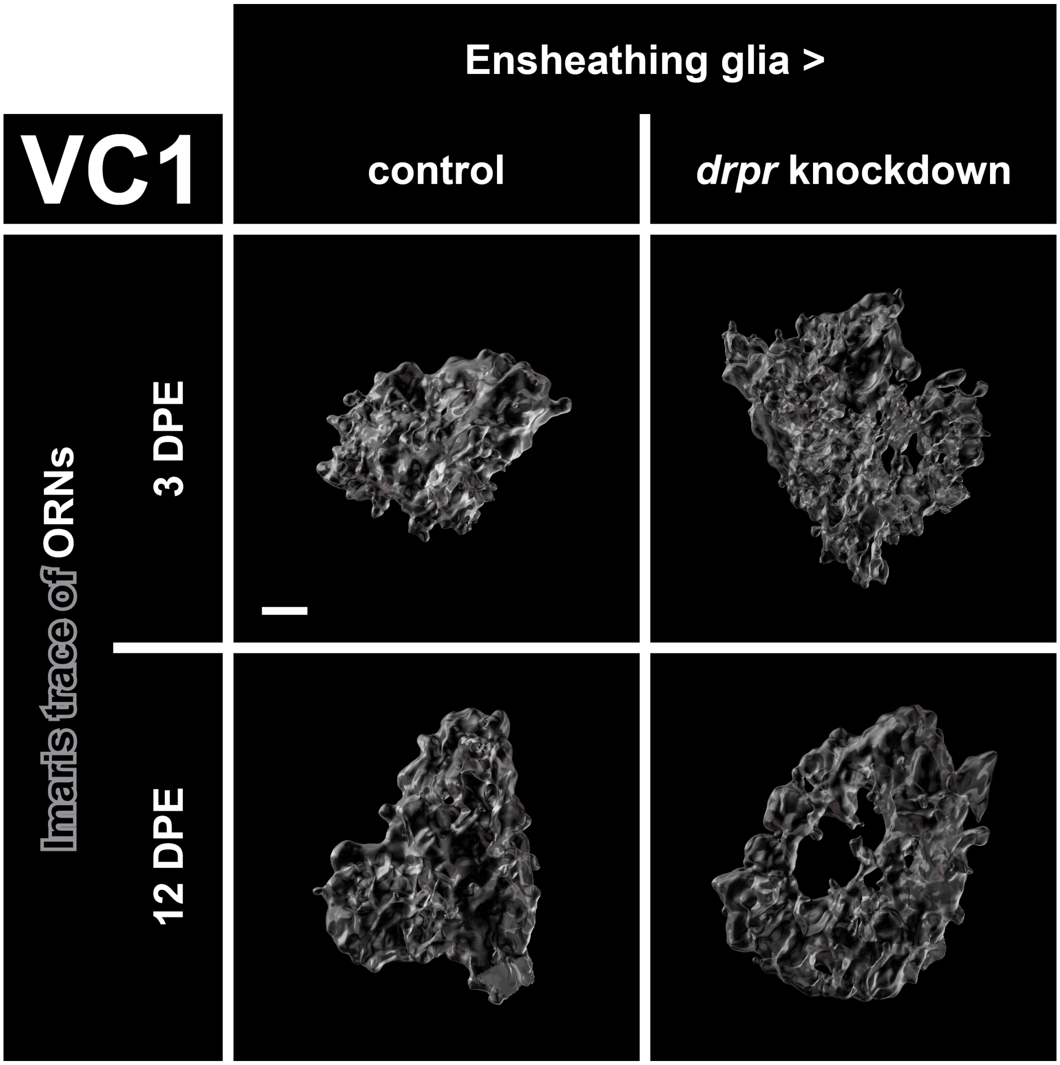

Supplementary Figure 1C. Loss of Draper in ensheathing glia leads to persistent increases in presynaptic content and terminal arbor size of VC1 ORNs. Surfaces of VC1 ORN terminal arbors recognized by Imaris (gray) corresponding to representative images shown in Figures 1F & 1G. Genotypes: control is +/Or85e-mCD8::GFP; +/MZ0709-Gal4. *drpr* knockdown is +/Or85e-mCD8::GFP; UAS-*drpr* RNAi/MZ0709-Gal4. Scale bar = 3 µm.

See "Video 2.MP4"

Supplementary Figure 1D. ("Video 2.MP4") Loss of Draper in ensheathing glia leads to persistent increases in presynaptic content and terminal arbor size of VC1 ORNs. Videos of Brp puncta inside ORN terminal arbors corresponding to representative images shown in Figures 1F & 1G. Genotypes: control is +/Or85e-mCD8::GFP; +/MZ0709-Gal4. *drpr* knockdown is +/Or85e-mCD8::GFP; UAS-*drpr* RNAi/MZ0709-Gal4.

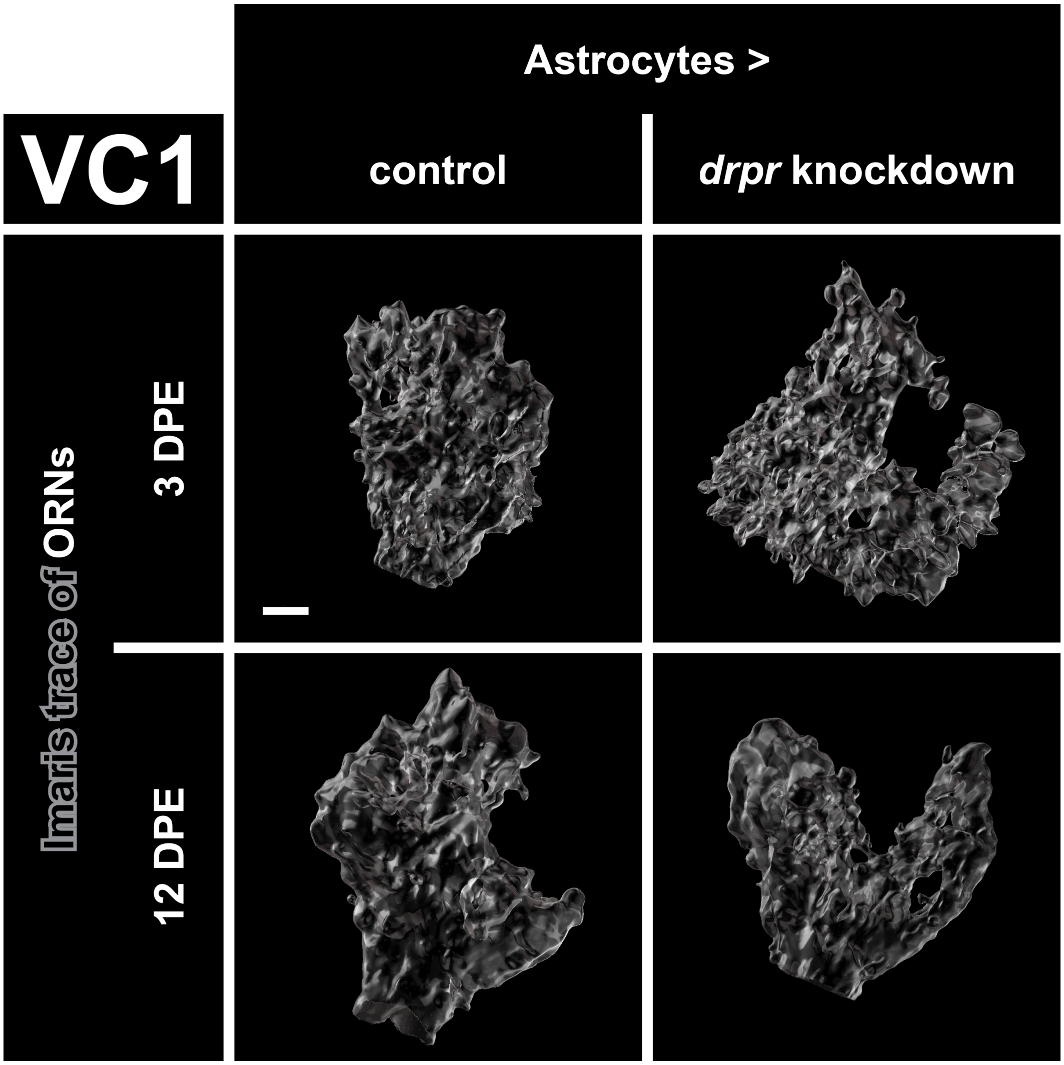

Supplementary Figure 2A. Loss of Draper in astrocytes does not alter VC1 ORN presynaptic content or terminal arbor size, but leads to transient changes in glomerular shape. Surfaces of VC1 ORN terminal arbors recognized by Imaris (gray) corresponding to representative images shown in Figures 2A & 2B. Genotypes: control is +/Or85e-mCD8::GFP; +/R86E01-Gal4. *drpr* knockdown is +/Or85e-mCD8::GFP; UAS-*drpr* RNAi/R86E01-Gal4. Scale bar = 3 μm.

See "Video 3.MP4"

Supplementary Figure 2B. ("Video 3.MP4") Loss of Draper in astrocytes does not alter VC1 ORN presynaptic content or terminal arbor size, but leads to transient changes in glomerular shape. Videos of Brp puncta inside ORN terminal arbors corresponding to representative images shown in Figures 2A & 2B. Genotypes: control is +/Or85e-mCD8::GFP; +/R86E01-Gal4. *drpr* knockdown is +/Or85e-mCD8::GFP; UAS-*drpr* RNAi/R86E01-Gal4.

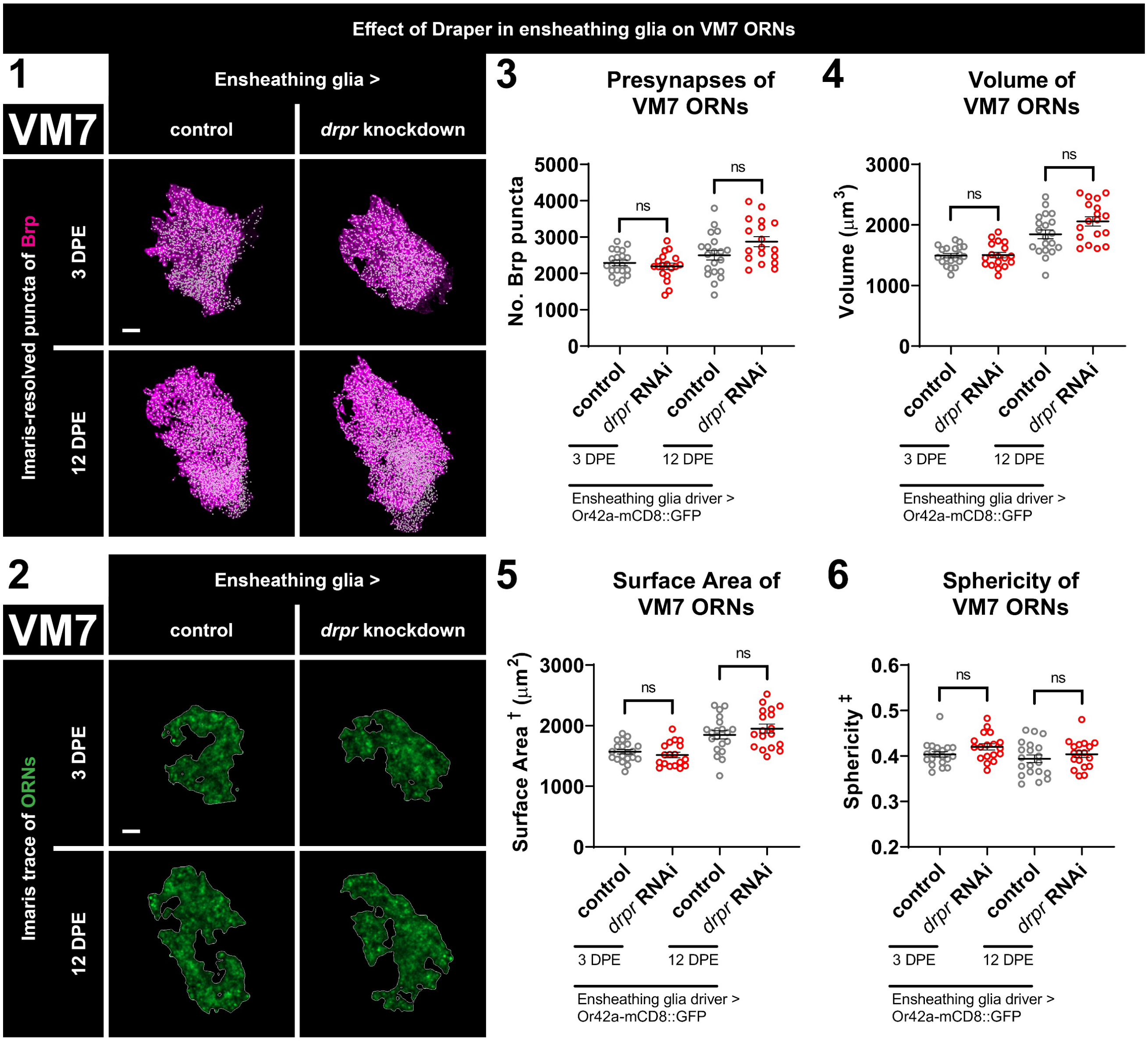
Effect of Draper in ensheathing glia on VM7 ORNs
1
VM7
Ensheathing glia >
control
drpr knockdown
Imaris-resolved puncta of Brp
3 DPE
12 DPE
3
Presynapses of VM7 ORNs
No. Brp puncta
ns
ns
control
drpr RNAi
control
drpr RNAi
3 DPE
12 DPE
Ensheathing glia driver >
Or42a-mCD8::GFP
4
Volume of VM7 ORNs
Volume (μm3)
ns
ns
control
drpr RNAi
control
drpr RNAi
3 DPE
12 DPE
Ensheathing glia driver >
Or42a-mCD8::GFP
2
VM7
Ensheathing glia >
control
drpr knockdown
Imaris trace of ORNs
3 DPE
12 DPE
5
Surface Area of VM7 ORNs
Surface Area † (μm2)
ns
ns
control
drpr RNAi
control
drpr RNAi
3 DPE
12 DPE
Ensheathing glia driver >
Or42a-mCD8::GFP
6
Sphericity of VM7 ORNs
Sphericity ‡
ns
ns
control
drpr RNAi
control
drpr RNAi
3 DPE
12 DPE
Ensheathing glia driver >
Or42a-mCD8::GFP

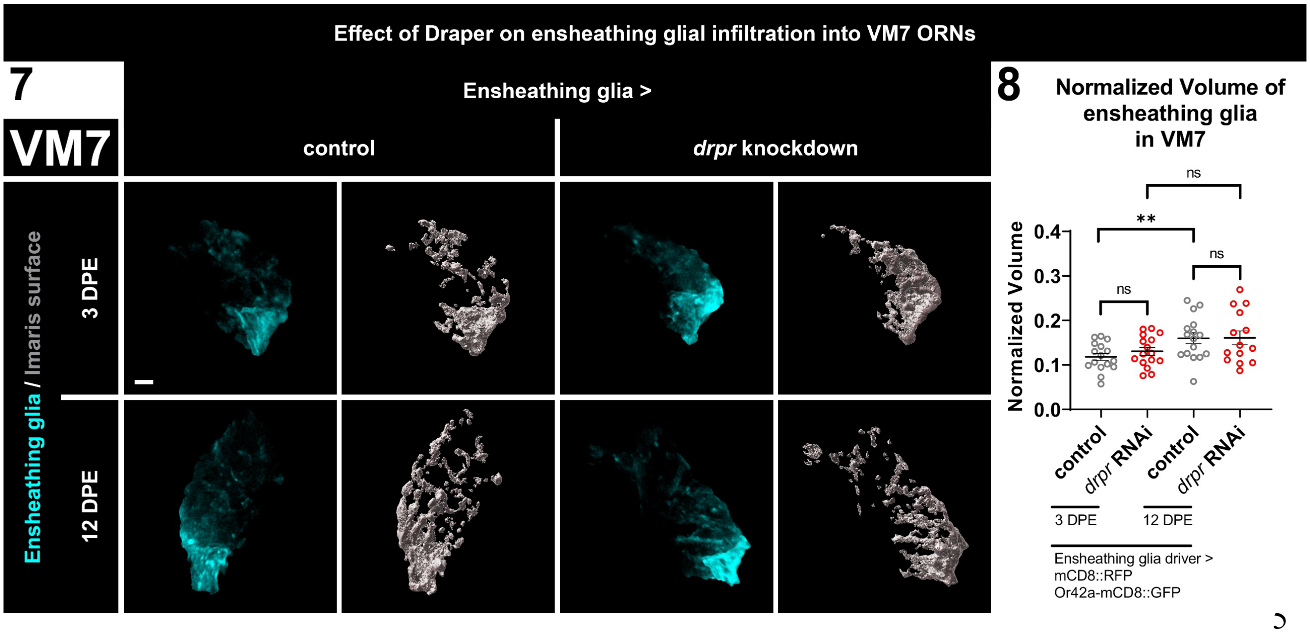
Effect of Draper on ensheathing glial infiltration into VM7 ORNs
7
VM7
Ensheathing glia >
control
drpr knockdown
Ensheathing glia / Imaris surface
3 DPE
12 DPE
8
Normalized Volume of ensheathing glia in VM7
Normalized Volume
ns
**
ns
ns
control
drpr RNAi
control
drpr RNAi
3 DPE
12 DPE
Ensheathing glia driver >
mCD8::RFP
Or42a-mCD8::GFP

Supplementary Figure 3A. Loss of Draper in ensheathing glia does not alter presynaptic content, terminal arbor size, or shape of VM7 ORNs. **(1)** MIP overlays of Brp stain (magenta) and recognized puncta (white), **(2)** single coronal slices of membrane staining (green) and traces (white) of pb1A ORN terminal arbors in control flies and flies with constitutive knockdown of *drpr* in ensheathing glia at 3 DPE and 12 DPE. Draper in ensheathing glia does not affect **(3)** presynapse number, **(4)** volume, **(5)** surface area, or **(6)** sphericity of VM7 ORN terminal arbors. **(7)** MIP of ensheathing glia processes (cyan) in the interior of VM7 ORN terminal arbors and recognized glial surface (gray) in control flies and flies with constitutive knockdown of *drpr* in astrocytes at 3 DPE and 12 DPE. **(8)** Volume of ensheathing glia inside VM7 ORN terminal arbors normalized to terminal arbor volume. Ensheathing glia infiltrate into VM7 at 3 DPE and 12 DPE. The position of VM7 at the medial edge of the antennal lobe results in incorporation of bright ensheathing glia processes at the edge of the mask. †Surface area is calculated based on light-level confocal microscopy measurements. ‡Sphericity is the ratio of the surface area of an equal-volume sphere to the surface area of an object and ranges from 0 to 1 (most spherical). For each condition, n > 16 antennal lobes from 8 brains. $^{**}p < 0.01$ and ns = not significant. All error bars represent mean ± SEM. See (section 2.4. "Statistical analysis") for details of statistical tests used. Genotypes: **(1-6)** control is +/Or42a-mCD8::GFP; +/MZ0709-Gal4. *drpr* knockdown & *drpr* RNAi is +/Or42a-mCD8::GFP; UAS-*drpr* RNAi/MZ0709-Gal4. **(7, 8)** control is UAS-mCD8::RFP/Or42a-mCD8::GFP; UAS-Luciferase/MZ0709-Gal4. *drpr* knockdown & *drpr* RNAi is UAS-mCD8::RFP/Or42a-mCD8::GFP; UAS-*drpr* RNAi/MZ0709-Gal4. Scale bar = 3 µm.

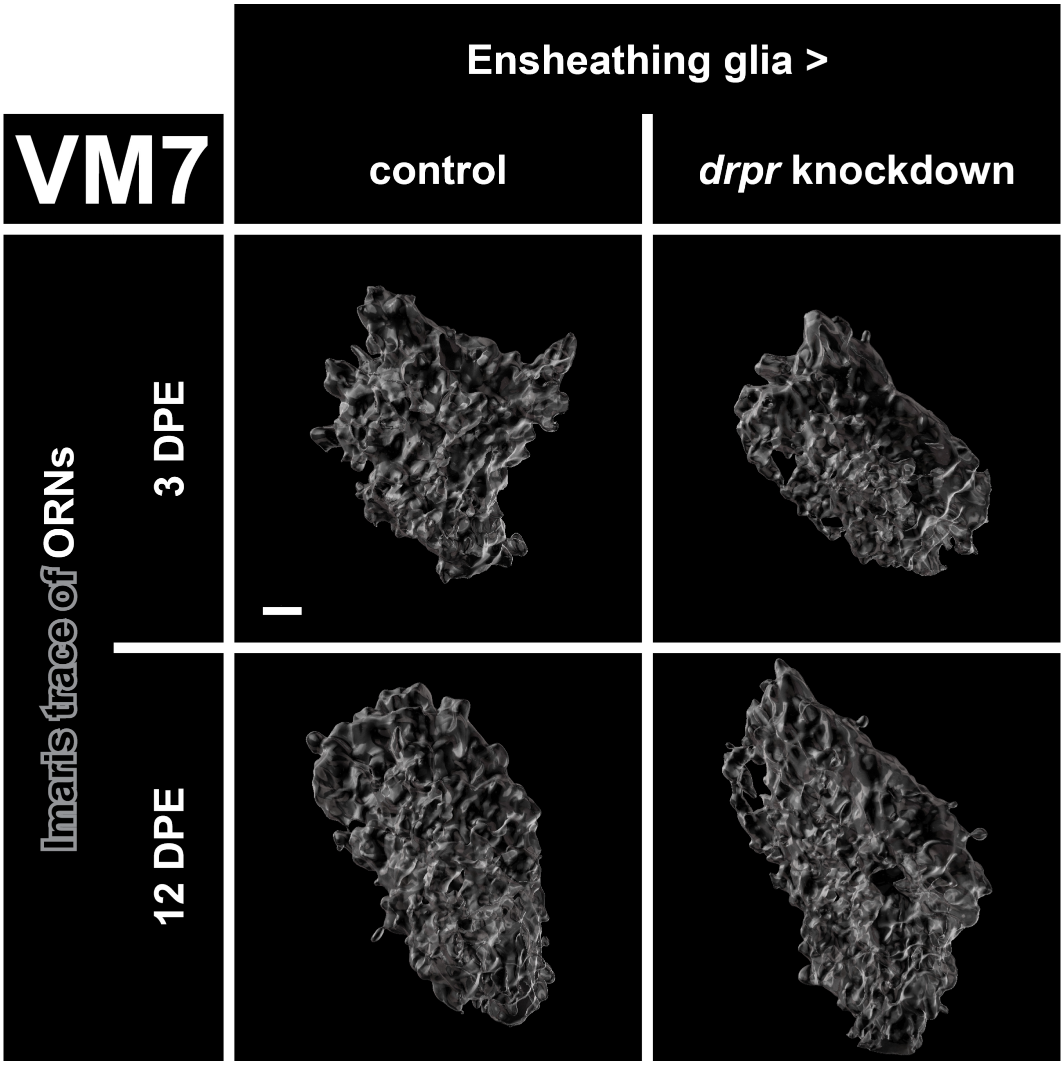

Supplementary Figure 3B. Loss of Draper in ensheathing glia does not alter presynaptic content, terminal arbor size, or shape of VM7 ORNs. Surfaces of VM7 ORN terminal arbors recognized by Imaris (gray) corresponding to representative images shown in Supplemental Figures 3A1 & 3A2. Genotypes: control is +/Or42a-mCD8::GFP; +/MZ0709-Gal4. *drpr* knockdown is +/Or42a-mCD8::GFP; UAS-*drpr* RNAi/MZ0709-Gal4. Scale bar = 3 µm.

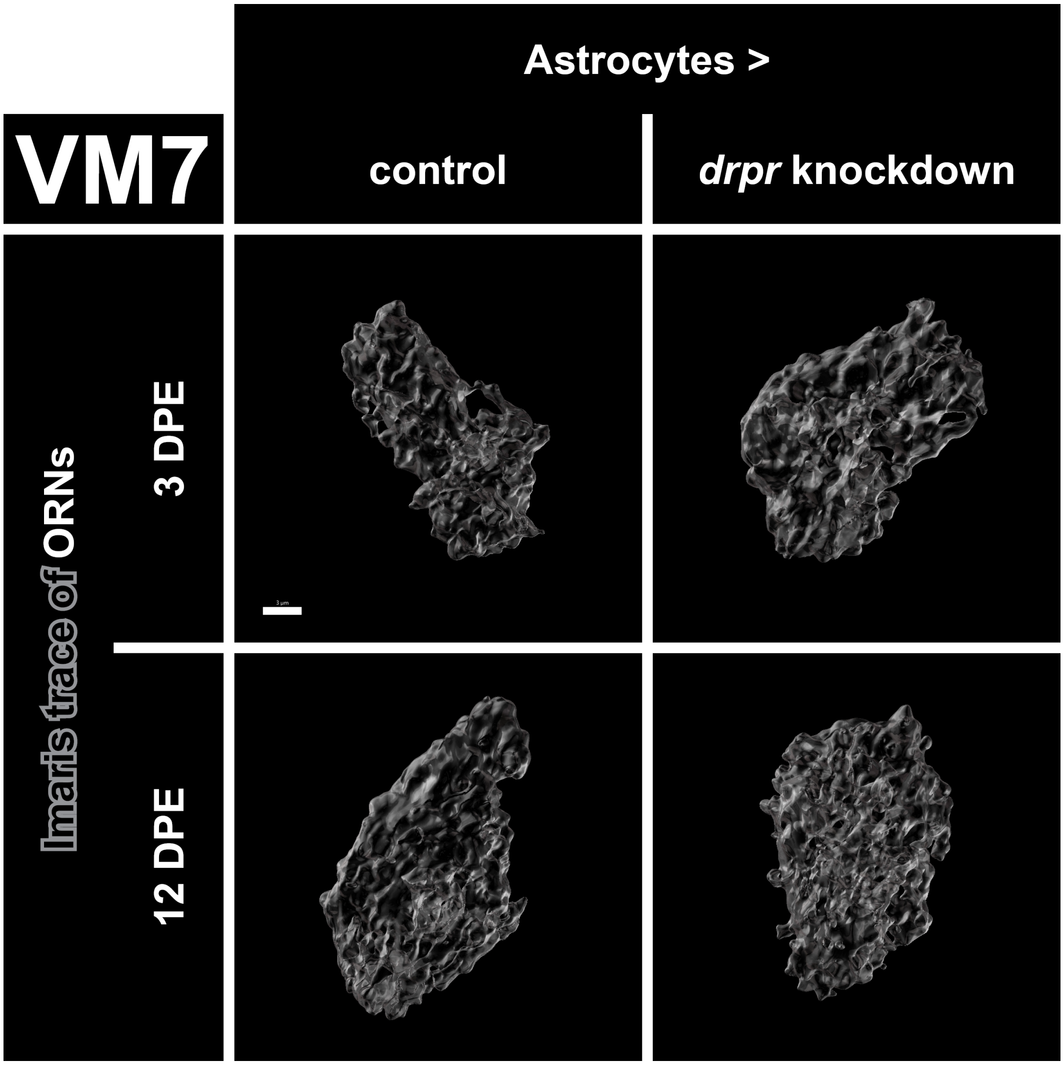

Supplementary Figure 4A. Loss of Draper in astrocytes results in transient increases in presynaptic content and terminal arbor size of VM7 ORNs. Surfaces of VM7 ORN terminal arbors recognized by Imaris (gray) corresponding to representative images shown in Figures 3A & 3B. Genotypes: control is +/Or42a-mCD8::GFP; +/R86E01-Gal4. *drpr* knockdown is +/Or42a-mCD8::GFP; UAS-*drpr* RNAi/R86E01-Gal4. Scale bar = 3 µm.

See "Video 4.MP4"

Supplementary Figure 4B. ("Video 4.MP4") Loss of Draper in astrocytes results in transient increases in presynaptic content and terminal arbor size of VM7 ORNs. Videos of Brp puncta inside ORN terminal arbors corresponding to representative images shown in Figures 3A & 3B. Genotypes: control is +/Or42a-mCD8::GFP; +/R86E01-Gal4. *drpr* knockdown is +/Or42a-mCD8::GFP; UAS-*drpr* RNAi/R86E01-Gal4.
